# Comparisons of treatment performance and therapy sequences in neuroendocrine neoplasms using progression-free survival ratios

**DOI:** 10.1007/s00259-025-07411-y

**Published:** 2025-06-21

**Authors:** Philipp Melhorn, Elisabeth Kretschmer-Chott, Peter Mazal, Markus Raderer, Barbara Kiesewetter

**Affiliations:** 1 Division of Oncology, Department of Medicine I, Medical University of Vienna, Waehringer Guertel 18–20, Vienna, A-1090 Austria; 2 Christian Doppler Laboratory for Personalized Immunotherapy, Department of Medicine I, Medical University of Vienna, Vienna, Austria; 3 Division of Nuclear Medicine, Department of Biomedical Imaging and Image-guided Therapy, Medical University of Vienna, Vienna, Austria; 4 Department of Pathology, Medical University of Vienna, Vienna, Austria

**Keywords:** Neuroendocrine tumors, Neuroendocrine carcinoma, Prognosis, Treatment, Effectiveness

## Abstract

**Introduction:**

Optimal sequencing of therapies is an important unresolved issue in metastatic neuroendocrine neoplasms (NEN). Progression-free survival (PFS) ratios constitute a potential method for intra-patient treatment comparisons and were used in this analysis to assess the relative treatment benefit of established therapies.

**Methods:**

This retrospective study included NEN patients of the Medical University of Vienna (treated in 2010–2024) who had metastatic disease and had received ≥ 2 palliative systemic therapies. The primary objective was the calculation of PFS ratios for therapy sequences, with the PFS ratio defined as the proportion of PFS2 (subsequent treatment) and PFS1 (prior treatment).

**Results:**

Of the 177 patients included, 104 had neuroendocrine tumors (NET) G1/G2, 16 NET G3, 28 lung/thymic carcinoids, and 29 a neuroendocrine carcinoma (NEC). In terms of treatment sequence, SSA was the most common first-line treatment in NET G1/G2 and thoracic carcinoids (*n* = 84), frequently followed by PRRT (*n* = 60), everolimus (*n* = 13), and treatments grouped as ‘other’ (*n* = 9). After platinum/etoposide in NEC (*n* = 26), FOLFOX/FOLFIRI (*n* = 13), CAPTEM (*n* = 3), and ‘other’ therapies (*n* = 9) were second-line therapies. The median PFS ratio for PRRT after first-line SSA was 1.86, for everolimus 0.99, and for ‘other’ treatments 0.59 (*p* = 0.004). Following platinum/etoposide, FOLFOX/FOLFIRI had a median PFS ratio of 0.46. In subgroup analyses according to primary localization, everolimus led to disproportionately long PFS intervals following SSA/PRRT in lung/thymic carcinoids, whereas therapies after PRRT exhibited relatively low PFS ratios in enteropancreatic NET.

**Conclusions:**

PFS ratios accommodate the heterogeneity of NEN and can provide insights regarding treatment performance and treatment sequencing.

**Supplementary Information:**

The online version contains supplementary material available at 10.1007/s00259-025-07411-y.

## Introduction

Current treatment algorithms for neuroendocrine neoplasms (NEN) are stratified according to several clinicopathologic factors such as primary site of disease and tumor grading, illustrating the great heterogeneity of this malignant disease [1–4]. Common sites of origin are the gastroenteropancreatic tract and the lungs, but NEN can occur throughout the body [5, 6]. Corresponding to the tumor aggressivity and disease pace, NEN are subdivided into three tumor grades (G1-G3) [7, 8]. Furthermore, a pathologic dichotomy exists between well-differentiated neuroendocrine tumors (NET G1-G3) and high-grade neuroendocrine carcinomas (NEC) [7, 9]. The somatostatin receptor (SSTR) is a cell surface target present in most NET [10], and SSTR expression is a prerequisite for certain antitumor therapies, i.e., somatostatin analogs (SSA) and peptide receptor radionuclide therapy (PRRT) [1].

In the metastatic setting, a broad array of treatments is available, ranging from SSTR-targeted therapies to small-molecule inhibitors and chemotherapy, with many patients receiving several lines of therapy over the disease course [1]. Given the often protracted overall survival (OS) in NET, generally low radiographic response rates observed with NET therapies, and confounding cross-over effects [11], progression-free survival (PFS) has been a preferred primary endpoint in NET trials [12, 13]. Furthermore, most pivotal trials in NET were placebo-controlled, e.g., CLARINET (SSA lanreotide) [14], PROMID (SSA octreotide) [15], RADIANT-3 and -4 (everolimus) [16, 17], and the pivotal trial on sunitinib (Raymond et al.) [18], while NETTER-1 (PRRT) [19] and NETTER-2 (PRRT) [20] had a control group with high-dose SSA. In contrast, these therapies are not approved for NEC, which are significantly more aggressive and require urgent chemotherapy, with platinum/etoposide as the standard first line and few options available thereafter [3]. To date, the optimal treatment sequence in NEN is largely unknown [1, 2] and represents one of the greatest challenges in clinical practice. Comparative data outside of the pivotal trials are currently limited. In addition, especially NET patients can exhibit very heterogeneous clinical courses, ranging from many years of indolent behavior in low-grade midgut NET to sometimes rapidly progressive disease in pancreatic NET with high liver tumor burden, making an objective comparison of available therapies difficult.

A potential solution to this challenge is the progression-free survival (PFS) ratio. More than 25 years ago, PFS ratios were proposed as a metric for phase II trials to quantify the clinical benefit of a tumor-stabilizing (rather than a cytotoxic) drug [21]. The PFS ratio compares PFS intervals within individual patients and is defined as the proportion of PFS2 (of the experimental/subsequent treatment) and PFS1 (of the antecedent treatment, e.g., the first-line therapy or the last standard treatment) [21, 22]. In the original concept proposal, an ratio of ≥ 1.33 was defined to be an excellent and unanticipated improvement [21]. Although PFS ratios have been increasingly used in precision oncology trials [22–25] and other studies [26–30], there are some methodological concerns, threshold values are varying, and the experience is still limited [31–36]. Nonetheless, by using each patient as his/her own control and thus accounting for the individual tumor biology, this measure could be an objective method to judge treatment performance.

In this study, we wanted to repurpose PFS ratios as a method to compare the relative tumor-stabilizing effect of established NEN treatments in order to inform current treatment sequencing practices and provide insights for future clinical trial design. We calculated the PFS ratios of various treatment sequences, e.g., different therapies following first-line SSA in NET and after first-line platinum/etoposide in NEC. Therapies with a high median PFS ratio could be considered “disproportionately effective”, and this might warrant use in earlier treatment lines.

## Methods

### Patients and treatments

This retrospective data analysis included patients with histologically verified NEN of any grade and site treated at the Medical University of Vienna, a European Neuroendocrine Tumor Society (ENETS) Center of Excellence, between January 2010 and January 2024. Patients were required to have ≥ 2 recorded palliative treatment lines, and adjuvant/neoadjuvant therapies were excluded. To ensure a clean therapy sequence data set, combination therapies, add-on therapies, maintenance therapies (i.e., SSA following PRRT), and treatments with prompt discontinuation (e.g., due to toxicity) or lack of relevant clinical data were excluded. Patient data were extracted from electronic health records, entered into a protected FileMaker database (Claris International Inc., Santa Clara, California, USA), and pseudonymized prior to analysis. Clinical data included baseline patient characteristics (e.g., age, sex, and Eastern Cooperative Oncology Group [ECOG] status), disease information (e.g., histology, primary tumor origin, disease setting, and functional status), and outcome data (e.g., tumor response, PFS, and OS). Moreover, the latest mortality data for the included patients were retrieved via the IT4Science service of the Medical University of Vienna/Statistics Austria. Exclusion criteria were missing clinical, histological, or radiological data. This analysis was approved by the Ethics Committee of the Medical University of Vienna (EK No: 1035/2024).

### Objectives

The main variable of interest was the PFS ratio (also called Growth Modulation Index), defined as the ratio of PFS2 (PFS of the subsequent treatment) and PFS1 (PFS of the antecedent treatment). The primary objective was to calculate the PFS ratios for certain therapy sequences (chosen based on the observed frequency) and subgroups. Based on these PFS ratios, treatment performances were estimated and compared. As a secondary objective, OS was analyzed based on tumor histology and primary tumor site and compared between patients receiving different therapy sequences. The initial data analysis included a descriptive analysis of the patient population and a breakdown of the PFS intervals according to treatment type and line. Furthermore, the correlation between PFS1 and PFS2 intervals was investigated. Response assessment was performed as per current guidelines at intervals of about 3–6 months, and disease progression was based on routine radiological reports. PFS was calculated from the treatment start date to disease progression or death, and patients were censored at the last follow-up date if no event had occurred. OS was defined as the interval from treatment start to the date of death.

### Statistical analysis

The statistical programming language R version 4.4.2 with the packages tidyverse [37], ggsurvfit [38], survival [39], networkD3 [40], lubridate [41], gtsummary [42], and gt [43] was used. Frequencies and percentages (for categorical variables) and ranges and medians (for quantitative variables) were used to statistically describe the patient population. Correlation between PFS2 and PFS1 was assessed graphically with scatterplots and numerically described with Spearman’s rank correlation coefficient (including only cases with progression on the second treatment) and with the R package SurvCorr [44] (for partially censored data). The Kaplan–Meier methodology was used for calculating the median PFS ratio in order to account for right-censoring (incomplete PFS2 interval and hence a potentially higher PFS ratio), as previously described [27]. PFS ratio distributions between subgroups were compared using the log-rank test. Similarly, the Kaplan–Meier method was used for OS calculations and the log-rank test to evaluate the difference in the distribution of time to all-cause death. Furthermore, a multivariable Cox regression was fitted to adjust comparisons of OS between treatments for grading, localization, and ECOG. For all tests, a two-tailed p-value < 0.05 was considered statistically significant, and no adjusting for multiple testing was performed due to the exploratory and hypothesis-generating nature of this analysis [45, 46].

## Results

### Baseline characteristics

Out of 500 NEN patients screened, 177 had two or more palliative systemic treatments and were eligible for the current analysis. Half of the patients (51%) were male, and the median age at diagnosis was 58 years (range: 25–82 years). Overall, 29 patients (16%) had NEC, 104 (59%) NET G1/G2, 16 (9%) NET G3, and 28 patients (16%) typical/atypical carcinoid (TC/AC, that is thoracic NET). The most frequent primary sites for NEC were pancreas (*n* = 12, 41%), colon/rectum (*n* = 8, 28%), and unknown (cancer of unknown primary, CUP, *n* = 5, 17%). Most NET G1/G2 were located in the small intestine (*n* = 44, 42%) and in the pancreas (*n* = 39, 38%), whereas NET G3 originated mainly from the pancreas (*n* = 9, 56%), and only few patients had small intestinal (*n* = 3, 19%) or CUP (*n* = 2, 13%) NET G3, see Table 1. Ki-67 index differed notably between the histologic subtypes, with 5% in NET G1/G2, 26% in NET G3, 75% in NEC, and 8% in TC/AC.

**Table 1:** Patient characteristics

Variable	*N*	Overall *N* = 177^*1*^	NEC *N* = 29^*1*^	NET G1/G2 *N* = 104^*1*^	NET G3 *N* = 16^*1*^	TC/AC *N* = 28^*1*^
**Sex**	177					
Female		87 (49%)	14 (48%)	52 (50%)	5 (31%)	16 (57%)
Male		90 (51%)	15 (52%)	52 (50%)	11 (69%)	12 (43%)
**Age at diagnosis**	177	58 (25, 82)	54 (26, 80)	59 (33, 82)	54 (25, 73)	67 (40, 81)
**Localization**	177					
Colon/rectum		13 (7.3%)	8 (28%)	5 (4.8%)	0 (0%)	0 (0%)
CUP		16 (9.0%)	5 (17%)	9 (8.7%)	2 (13%)	0 (0%)
Lung/thymus		28 (16%)	0 (0%)	0 (0%)	0 (0%)	28 (100%)
Other		13 (7.3%)	4 (14%)	7 (6.7%)	2 (13%)	0 (0%)
Pancreas		60 (34%)	12 (41%)	39 (38%)	9 (56%)	0 (0%)
Small intestine		47 (27%)	0 (0%)	44 (42%)	3 (19%)	0 (0%)
**Ki-67 index**	153	10 (0, 97)	75 (22, 97)	5 (1, 19)	26 (21, 50)	8 (0, 20)
**ECOG performance score**	153					
0		141 (92%)	25 (96%)	82 (94%)	16 (100%)	18 (75%)
1		10 (6.5%)	1 (3.8%)	4 (4.6%)	0 (0%)	5 (21%)
2		2 (1.3%)	0 (0%)	1 (1.1%)	0 (0%)	1 (4.2%)
**Functional symptoms**	174	48 (28%)	0 (0%)	40 (39%)	4 (25%)	4 (14%)
**Disease setting at diagnosis**	177					
Distant metastases		124 (70%)	23 (79%)	80 (77%)	13 (81%)	8 (29%)
Localized		32 (18%)	2 (6.9%)	14 (13%)	2 (13%)	14 (50%)
Locally advanced		21 (12%)	4 (14%)	10 (9.6%)	1 (6.3%)	6 (21%)
**Distant metastasis at any time point**	177	177 (100%)	29 (100%)	104 (100%)	16 (100%)	28 (100%)
**Surgery**	177	95 (54%)	5 (17%)	66 (63%)	6 (38%)	18 (64%)
**Number of treatments**	177	2 (2, 8)	2 (2, 4)	2 (2, 8)	3 (2, 4)	2 (2, 6)

^*1*^n (%); Median (Min, Max)

The ECOG performance status was excellent in most patients at diagnosis (ECOG 0 in 92%). In total, 28% of patients had functional symptoms, but this varied widely between NEC (0%), NET G1/G2 (39%), NET G3 (25%), and TC/AC (14%). The majority of patients with digestive NET/NEC had distant metastases at diagnosis (around 80%) and to a lesser extent localized (7–13%) and locally advanced disease (6–14%), while only 29% (8/28) of patients with lung/thymus NET were metastatic at diagnosis and 50% (*n* = 14) presented in a localized setting. All patients developed metastases during the disease course. In total, 95 patients (54%) had surgery. The median number of treatments was 2 (range: 2–8).

### Treatment lines

**Fig. 1:**
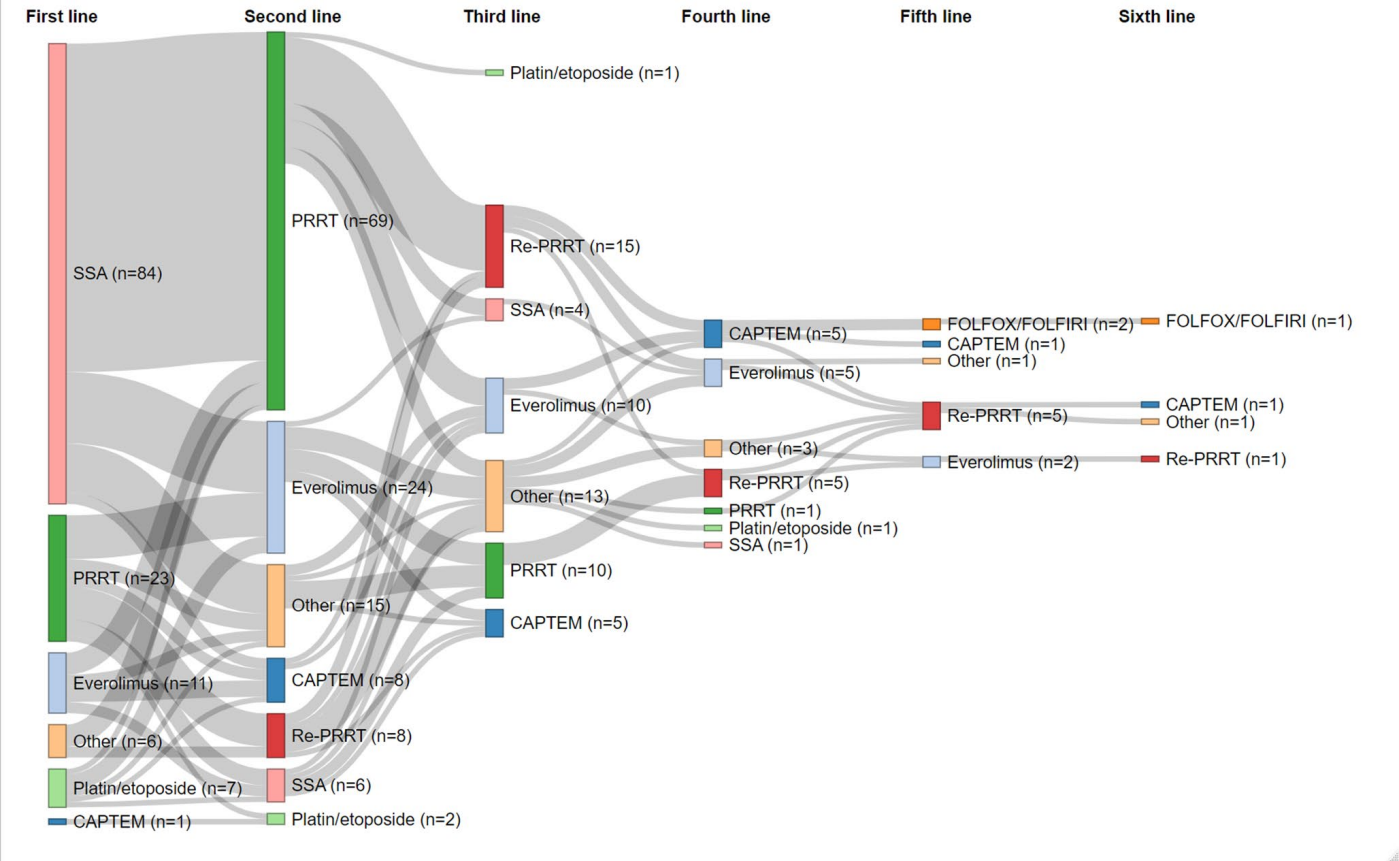
Sankey diagram of treatment sequencing in the NET G1/G2 and TC/AC subset

A total of 485 palliative treatments were recorded, 114 PRRT (96% had [^177^Lu]Lu-DOTA-TATE), 98 SSA, 59 everolimus, 44 capecitabine/temozolomide (CAPTEM), 44 cis- or carboplatin/etoposide, 38 Re-PRRT, 22 5-fluorouracil/leucovorin/oxaliplatin (FOLFOX) or 5-fluorouracil/leucovorin/irinotecan (FOLFIRI), and 66 therapies referred to herein as ‘other’, see Table S1 for a breakdown by histology and for further information regarding therapy protocols. The most common treatments in NEC were chemotherapies such as platinum/etoposide (*n* = 27), FOLFOX/FOLFIRI (*n* = 15), and CAPTEM (*n* = 8). SSTR-targeted treatments (86 PRRT, 83 SSA, 33 Re-PRRT) were frequently employed in NET G1/G2 besides everolimus (*n* = 34). In TC/AC, everolimus (*n* = 18) and CAPTEM (*n* = 13) were favored treatments. A high variance in the duration of PFS intervals was observed, see Figure S1. With regards to therapy sequences in NET G1/G2 and TC/AC, first-line SSA (*n* = 84) was frequently followed by PRRT (*n* = 60), everolimus (*n* = 13), and ‘other’ therapies (*n* = 9), see Fig. 1. In several cases, upfront PRRT (*n* = 23) was followed by everolimus (*n* = 8) or Re-PRRT (*n* = 6). In NET G3, there was no particularly frequent sequence, see Figure S2A. After platinum/etoposide in NEC (*n* = 26), FOLFOX/FOLFIRI (*n* = 13), CAPTEM (*n* = 3), and ‘other’ therapies (*n* = 9) were applied second-line therapies, see Figure S2B.

### PFS intervals

As a crude comparison of first-line palliative treatments, the median PFS for SSA (*n* = 84) in patients with NET G1/G2 or TC/AC was 15.1 months (95% CI 11.7–20.3 months), for PRRT (*n* = 23) 17.4 months (95% CI 10.9–56.2 months), and for everolimus (*n* = 11) 15.9 months (95% CI 8.0–not estimable [NE] months), see Figure S3. For CAPTEM (*n* = 6) in NET G3, the median PFS was 11.5 months (95% CI 3.7–NE months), whereas it was 6.0 months (95% CI 4.2–8.1 months) for platinum/etoposide in NEC (*n* = 26). In NET G1/G2 and TC/AC, the median PFS for second-line PRRT (*n* = 69) was 28.3 months (95% CI 18.7–39.8 months), while it was much shorter for the other second-line treatments, with 8.0 months (95% CI 5.7–19.5 months) for everolimus (*n* = 24), 13.5 months (95% CI 10.7–NE months) for CAPTEM (*n* = 8), and 8.3 months (95% CI 5.6–27.2 months) for ‘other’ treatments (*n* = 15), see Figure S4. In the second line in NEC, the median PFS was 2.8 months (95% CI 2.1–7.4 months) for FOLFOX/FOLFIRI (*n* = 14), 5.2 months (95% CI 3.4–NE months) for CAPTEM (*n* = 5), and 2.3 months (95% CI 1.8–NE months) for ‘other’ treatments (*n* = 9). The most common second-line treatment in NET G3 was also CAPTEM (*n* = 6), exhibiting a median PFS of 10.5 months (95% CI 7.7–NE months).

### Correlation of PFS intervals

**Fig. 2:**
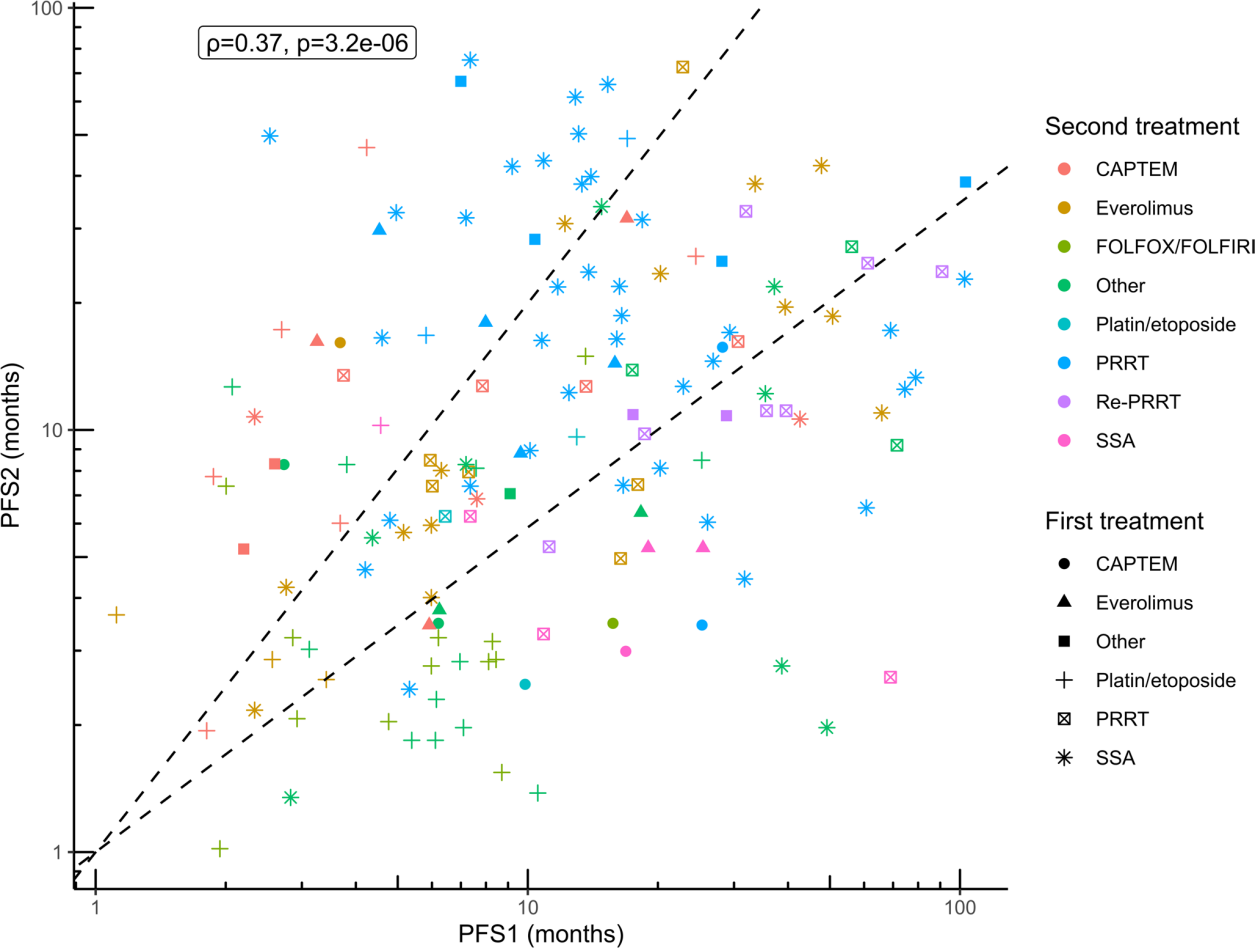
Correlation between PFS1 and PFS2 with *n* = 150 and log-10 scale for both axes, showing areas with high (> 1.3, top left) and low (< 0.77, bottom right) PFS ratios

Spearman correlation between the PFS of the first-line treatment (PFS1) and the PFS of the second-line treatment (PFS2) for all patients irrespective of histology who were progressive in the second line (*n* = 150) was weak to moderate [48] (ρ = 0.37, *p* < 0.001), meaning that PFS1 may not be able to accurately predict PFS2, see Figure S5. For the total cohort (*n* = 177) with partially censored survival times, the correlation coefficient was estimated at ρ = 0.35 (95% CI 0.21–0.48), see Figure S6. An L-shaped distribution of PFS intervals was noted, with some patients having either a relatively long PFS1 or PFS2 interval (over 3–5 years). When segmenting the PFS1-PFS2 scatterplot into areas with a high (> 1.3) or low (< 0.77) PFS ratio, second-line PRRT seemed to frequently elicit a disproportionately high relative treatment benefit, e.g., compared to everolimus, see Fig. 2. Likewise, CAPTEM resulted in longer PFS2 compared to FOLFOX/FOLFIRI. In addition, Re-PRRT after PRRT generally led to shorter PFS2 than PFS1.

### PFS ratios

**Fig. 3:**
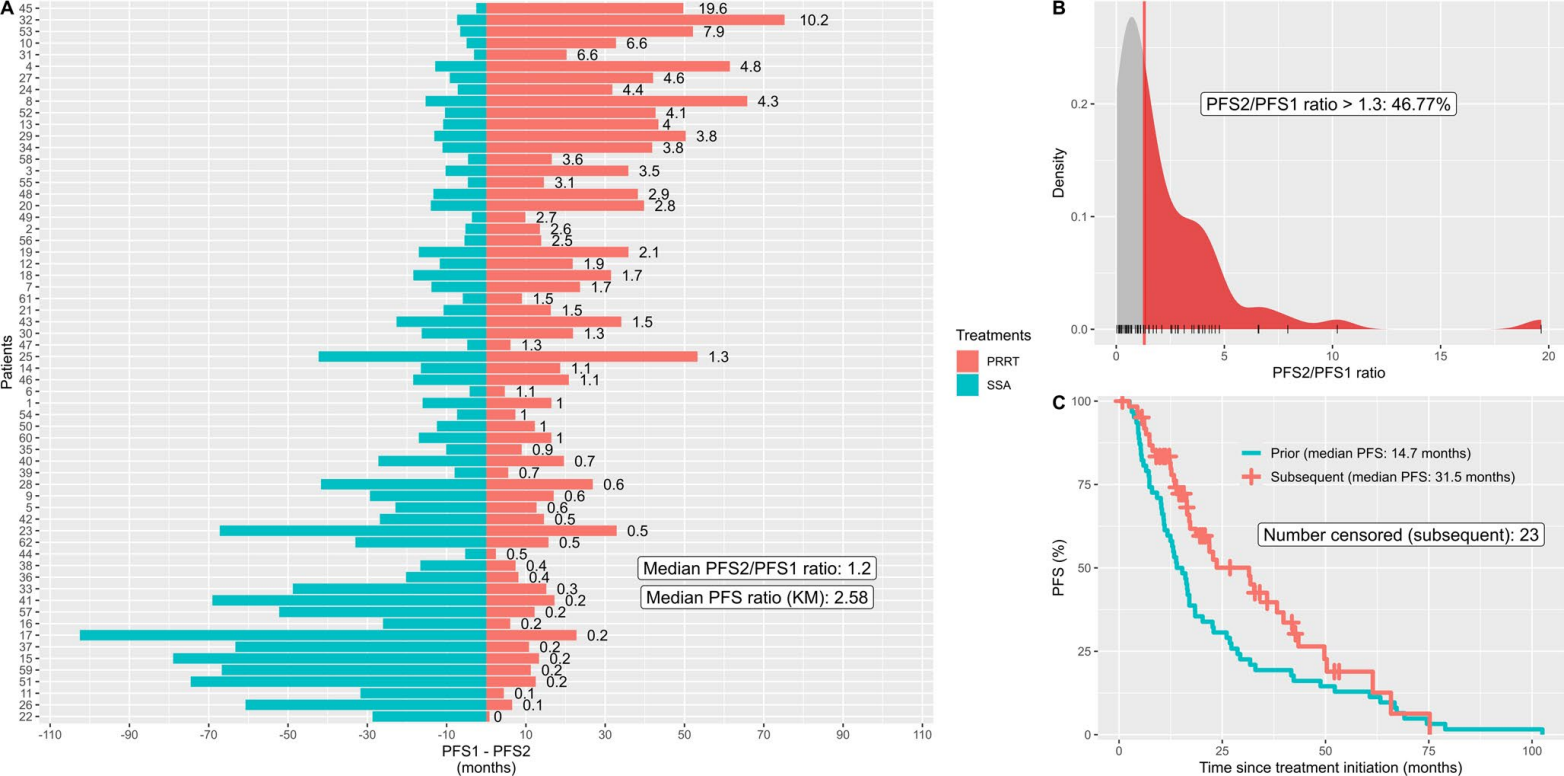
PFS ratios for the sequence SSA to PRRT in individual patients with NET G1/G2 or TC/AC (**A**), the distribution of these PFS ratios (**B**), and a Kaplan-Meier (KM) plot showing survival curves for the previous and subsequent treatment (**C**)

First, PFS ratios were calculated for therapy sequences across all treatment lines. Irrespective of censoring of PFS2, the median PFS ratio for the most frequent sequence in NET G1/G2 and TC/AC, SSA to PRRT, was 1.2, and thus almost half of the patients (47%) showed a disproportionately longer PFS2 duration (PFS ratio > 1.3), see Fig. 3. Following SSA in NET G1/G2 and TC/AC, everolimus showed similar PFS intervals, with a median PFS ratio of 0.99 and only 13% having a PFS ratio > 1.3, see Figure S7. In patients with NET G1/G2 or TC/AC receiving everolimus after PRRT, the median PFS ratio was low (0.41, 15% >1.3), see Figure S8. The median PFS ratio for FOLFOX/FOLFIRI after platinum/etoposide in NEC was low (0.46, 8% >1.3), while CAPTEM achieved mostly high PFS ratios in selected patients with NET G3 or NEC (median of 4.14 in NET G3 and 1.63 in NEC), see Figures S9 and S10. When determining median PFS ratios using the Kaplan–Meier (KM) method, PRRT after SSA showed a markedly higher median PFS ratio (2.58 versus 1.2) on account of the high number of censored patients (*n* = 23), whereas the results for the other sequences remained essentially unchanged (except for the sequence PRRT to everolimus with 0.58 versus 0.41), see Figs. 3 and S7-S10.

**Fig. 4:**
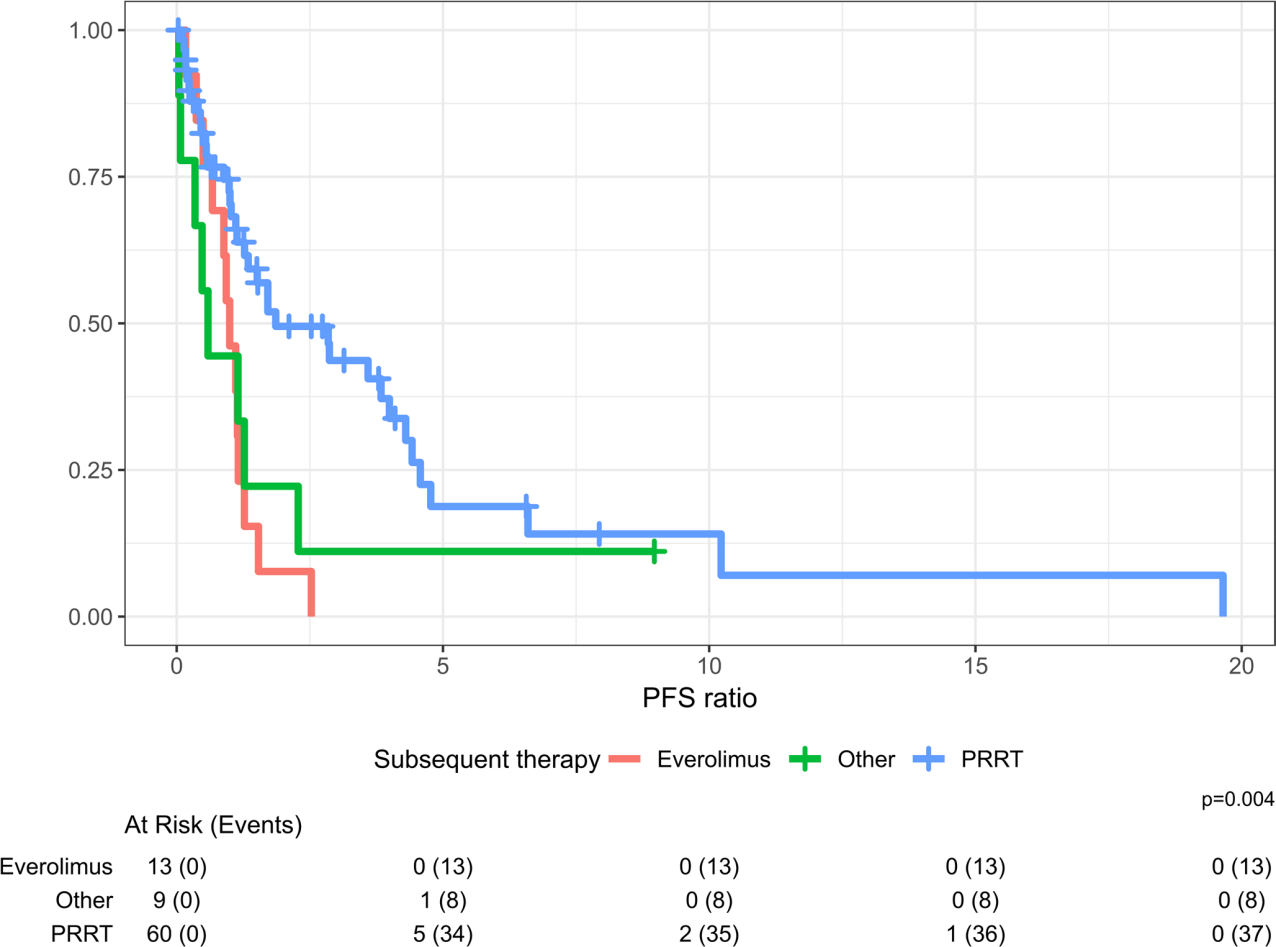
Distribution of PFS ratios of subsequent therapies following first-line SSA treatment in NET G1/G2 or TC/AC

Next, PFS ratios only for therapies after first-line SSA were computed, see Fig. 4. The median PFS ratio (KM) for PRRT after SSA was 1.86 (95% CI 1.27–4.3), for everolimus 0.99 (95% CI 0.67–NE), and for ‘other’ treatments 0.59 (95% CI 0.34–NE), with a significant difference in the distribution of these ratios (*p* = 0.004). This indicates that PRRT was associated with higher relative treatment benefit than everolimus.

**Fig. 5:**
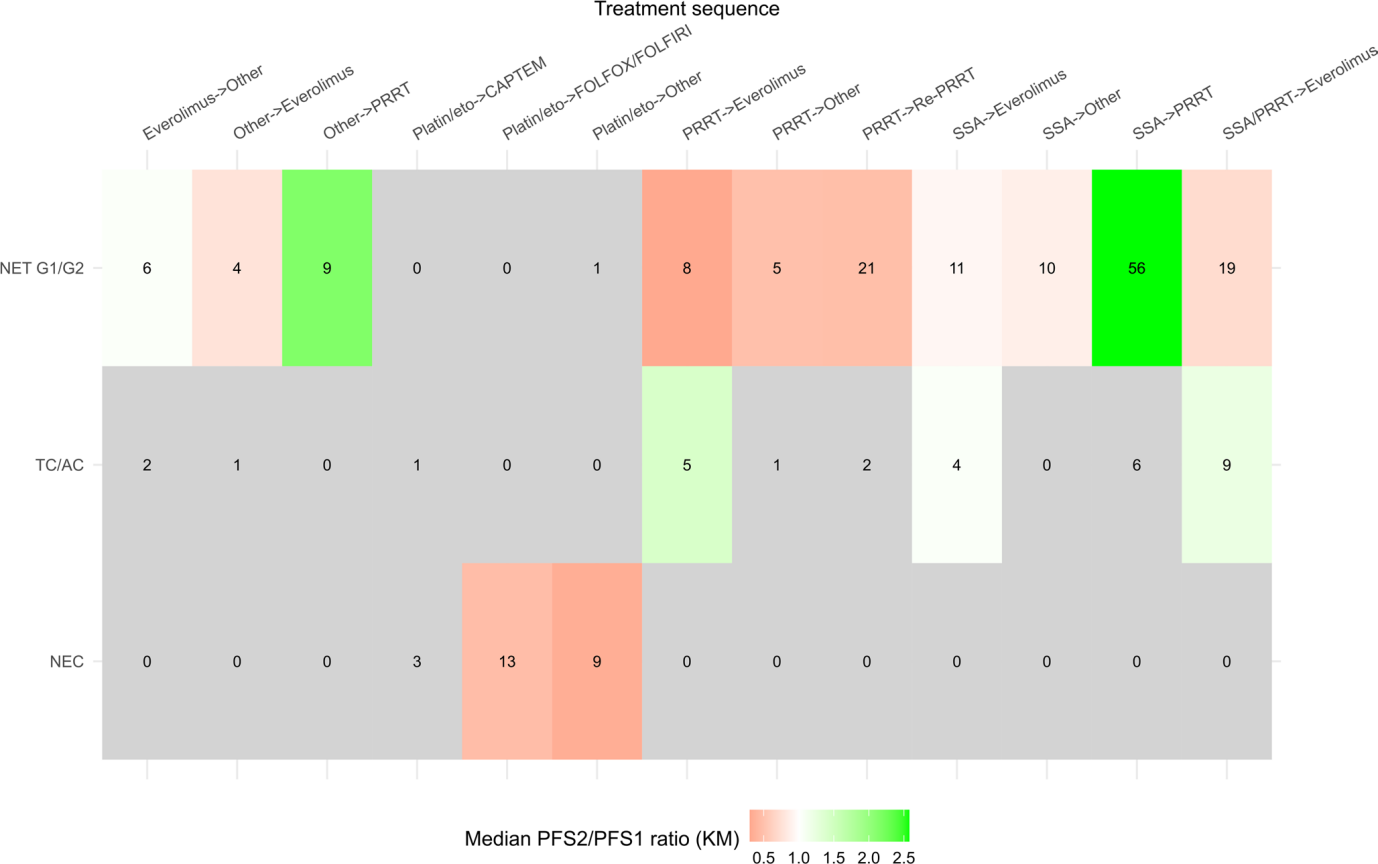
Heatmap showing the median PFS ratios (KM) for various treatment sequences according to the subgroups. The numbers in the tiles represent the sample size. Only ratios based on at least 4 measurements shown

Furthermore, PFS ratios (KM) were evaluated based on the three histologic subtypes (NET G1/G2, TC/AC, and NEC, see Fig. 5. In NET G1-G2, therapies after PRRT could not achieve a disproportionately long PFS2 (median of 0.30 for everolimus, 0.48 for ‘other’, and 0.47 for Re-PRRT). In contrast, the median PFS ratio (KM) for SSA to PRRT was 2.58 and for ‘other’ to PRRT 2.15. Discordant trends were observed regarding everolimus, as everolimus appeared to have superior outcomes after SSTR-targeted treatment in thoracic carcinoids (1.22) compared to (mainly enteropancreatic) NET G1-G2 (0.71). In particular, this difference was caused by PRRT (median PFS ratio of 1.43 for PRRT to everolimus in carcinoids), with either PRRT performing worse in TC/AC or everolimus being disproportionately superior in thoracic NET. In comparison, median PFS ratios for therapies after platinum/etoposide in NEC were relatively low (‘other’ treatments 0.34).

### Overall survival

The median OS from first-line start was 87.4 months (95% CI 69.4–100.6 months) in NET G1/G2 (*n* = 104), 37.8 months (95% CI 21.7–NE months) in NET G3 (*n* = 16), 13.1 months (95% CI 10.9–19.5 months) in NEC (*n* = 29), and 42.7 months (95% CI 31.9–NE months) in TC/AC (*n* = 28), amounting to a significant difference in OS (p < 0.001), see Figure S11. To evaluate potential differences in OS based on the treatment sequence, OS from second line start was calculated for PRRT, everolimus, and ‘other’ therapies following SSA in NET G1/G2 and TC/AC. There was no significant OS difference between the second lines (p = 0.12), with the median OS for everolimus being 48.7 months (95% CI 23.5–NE months, *n* = 13), for PRRT 40.4 months (95% CI 34.0–NE months, *n* = 60), and for ‘other’ treatments 21.4 months (95% CI 12.9–NE months, *n* = 9), see Figure S12. In a multivariable Cox regression, treatment sequence was also not significantly associated with OS, see Figure S13.

## Discussion

For NEC and NET, several treatment options are available. However, the relative clinical benefit and the sequencing of these therapies are currently unclear and are the subject of ongoing clinical trials. Limited published evidence on this matter comes from studies like SEQTOR [49], OCLURANDOM [50], and MGMT-NET [51]. The purpose of this analysis was to study PFS ratios in a large NEN collective (177 patients with 485 treatments in total) to estimate the relative growth-modulating effects of common treatment strategies for NEN. We hypothesized that these insights could serve as a foundation for devising informative clinical trials. In brief, based on the PFS ratios identified in the current analyses, PRRT showed outstanding effectiveness in enteropancreatic NET, while everolimus had superior disease-stabilizing activity in lung NET compared to PRRT/SSA. In contrast, and in line with the literature [3], therapies after platinum/etoposide in NEC yielded comparatively poor results.

The heterogeneity of neuroendocrine neoplasms and the diversity of available treatments make the interpretation and comparison of reported treatment outcomes exceedingly difficult. Randomized clinical trials are the gold standard for treatment evaluation, but they are time- and cost-intensive and limited to a few specific research questions [52]. They are designed for the explicit purpose to obtain drug approval—however, after approval, relevant clinical questions are often only answered in academic studies, if at all, at great expense. Conversely, even for a rare cancer such as NEN, there are ample retrospective data available, and PFS ratios allow for intra-patient comparisons that encompass a wide range of therapies and acknowledge the distinct tumor biology of each individual patient [35]. Previously, these PFS ratios were mainly used in precision oncology trials where a diverse array of cancers and treatments—similar to neuroendocrine neoplasms—was assessed [22–25].

Our research provides substantial evidence regarding the relative effectiveness of NEN therapies. To have a representative number of patients for each treatment, we focused on the established treatment options such as PRRT, SSA, and everolimus in NET. The median PFS ratio for PRRT after (first-line) SSA was 1.86, which indicates disproportionate effectiveness of PRRT compared to SSA and is much higher than the ratio 0.99 for everolimus and 0.59 for ‘other’ treatments after SSA.

In view of the dearth of comparative efficacy data, these results indicate that PPRT is a highly efficient treatment option, even though it was not associated with a significant OS difference compared to everolimus or other therapies in this study. Looking at prospective PFS data, PRRT-treated patients enrolled in NETTER-1 (HR 0.21, 95% CI 0.13–0.33) [19] and NETTER-2 (HR 0.276, 95% CI 0.182–0.418) [20] had an 72.4–79% lower risk of disease progression or death compared to (high-dose) SSA, and in NETTER-2 the median PFS was approximately 2.68 times longer (22.8 versus 8.5 months). In comparison, the median PFS ratio for PRRT versus prior SSA was 2.58 across all lines in our analysis. Data on everolimus from RADIANT-3 (HR 0.35, 95% CI 0.27–0.45, median PFS for everolimus 11.0 months versus 4.6 months with placebo) [16] and RADIANT-4 (HR 0.48, 95% CI 0.35–0.67, median PFS 11.0 months versus 3.9 months) [17] showed similar efficacy as for sunitinib (HR 0.42, 95% CI 0.26–0.66, median PFS 11.4 versus 5.5 months with placebo) [18]. In the OCLURANDOM trial in pancreatic NET, the median PFS was 20.7 in the PRRT arm versus 11 months again in the sunitinib arm [50]. Correspondingly, in a retrospective study of PRRT in pancreatic NET patients (NETTER-R), the median PFS was 24.8 months [53]. Though these are just indirect comparisons spanning different NET disease subpopulations, everolimus and sunitinib seem equally effective (roughly 11 months), whereas PRRT had a median PFS almost twice as long. This is in line with our data, i.e., a median PFS ratio of 0.58 of everolimus after PRRT, suggesting that everolimus might be associated with a PFS only about half as long as PRRT in many patients. Further evidence for the effectiveness of PRRT comes from a retrospective study called SeqEveRIV, which compared PRRT followed by everolimus with the reverse sequence in metastatic NET and showed better PFS with PRRT (median PFS of 24.5 vs. 16.1 months), albeit overall PFS for the two sequences were comparable [54]. Similarly, in a 2022 cohort study of 508 enteropancreatic NET patients with disease progression on first-line SSA, PRRT was associated with superior PFS as compared to chemotherapy or targeted therapy in both the unmatched and propensity score-matched population (median PFS of 2.5 vs. 0.7 years and 2.2 vs. 0.6 years, respectively) [55]. Crucially, however, PRRT is limited by the requirement for adequate SSTR expression, which may affect both patient selection and treatment performance. With regards to the superior effectiveness of everolimus over PRRT observed in lung NET, there are data from three large retrospective studies that might support inferior outcomes with PRRT in lung NET compared to enteropancreatic NET (median PFS of 20 vs. 30 months [56], 11 vs. 20–22 months [57], and 17.6 vs. 19.8–31.3 months [58]).

Previously, two network meta-analyses have attempted cross-trial comparisons based on a similar selection of NET trials [59, 60]. The first one found the lowest hazard for progression in gastrointestinal NET with PRRT + SSA (P score 0.97), followed by bevacizumab + SSA (0.68), interferon + SSA (0.59), everolimus + SSA (0.53), interferon (0.50), SSA (0.38), everolimus (0.33), and placebo (0.02) [59]. According to the second study, PRRT + SSA again had the highest probability (99.6%) of having the longest PFS, followed by sunitinib (64.5%), interferon + SSA (53%), SSA (46.6%), bevacizumab + SSA (45%), everolimus +/- SSA (33.6%), and placebo (7.6%) [60]. These analyses, however, have several weaknesses like study heterogeneity and varying trial quality and do not necessarily reflect current guidelines [61]. Hence, one might assume that the PFS ratios presented in this work are more robust estimates of relative treatment performance in the context of absent comparative trial data. Collectively, the results of those meta-analyses suggested that combination therapies have superior efficacy, however, with the exception of PRRT + SSA, these therapies are not standard practice as per current guidelines [1, 2]. In contrast to those data, our results suggested that everolimus had a similar effectiveness as SSA (median PFS ratio of 0.99) or even a higher treatment benefit, as it was mostly a post-SSA treatment (with theoretically lower ratios for the same sequence in later lines due to more advanced disease). In accordance with those results, PRRT seemed to be the most effective therapy in our analysis.

Sequencing of therapies is challenging and might become an even more important issue for the future given emerging treatment options like cabozantinib [62]. To date, most NET trials have been placebo-controlled, and there is an unmet need for more trials to compare active agents. Relevant studies currently ongoing include COMPETE (NCT03049189, PRRT versus everolimus in gastroenteropancreatic NET), COMPOSE (NCT04919226, PRRT versus everolimus/CAPTEM/FOLFOX in gastroenteropancreatic NET G2/G3), NCT05247905 (PRRT versus CAPTEM in pancreatic NET), LEVEL (NCT05918302, PRRT versus everolimus in lung/thymus NET), and NCT04665739 (PRRT versus everolimus in lung NET). Our findings underline the need for further head-to-head treatment comparisons, particularly considering different treatment lines and distinct primary site subgroups.

The limitations of this study pertain primarily to the methodological concerns regarding PFS ratios. First, the correlation of the PFS intervals has been discussed in the statistical literature as a prerequisite for efficient clinical trials [31]. The rather low correlation coefficient of 0.35 seen in this analysis is likely a result of different disease dynamics and treatment sequences that lead to substantial variations in PFS durations. Furthermore, a large variance in PFS intervals might cause unstable PFS ratios. Second, there was a selection bias as only patients with two or more treatment lines were included, and hence patients with a poor prognosis might benefit from different treatment strategies. Moreover, several disease characteristics influence treatment decisions, which can have a varying impact on survival outcomes. Third, some NET show an increase in tumor grade over time and might evolve into a more aggressive histology, which seems to occur particularly with PRRT [63] or alkylating agents [64] according to current evidence; accelerated tumor growth could limit the PFS of subsequent therapies (PFS2) and might result in smaller PFS ratios (and lower apparent effectiveness), which is not accounted for. Fourth, the generalizability of these results is limited by the retrospective nature, the single-center design, and the small sample size of certain subgroups, and multi-center efforts may provide more general estimates and elucidate different sequences. Nevertheless, uniform data collection and a “clean” sequence data set represent key assets of this analysis. Fifth, effectiveness was the only variable of interest here, but it is also important for future work to contrast the toxicity profile of these therapies.

To conclude, our study provides objective evidence for two interrelated issues, namely treatment performance and treatment sequencing. In this analysis, we have compared different treatments in NEN based on PFS ratios. Of most relevance for clinicians, our data showed that PRRT had a higher relative effectiveness than everolimus when compared to prior SSA in enteropancreatic NET, whereas everolimus was associated with better PFS compared to SSTR-targeted therapies in lung NET.

## Electronic supplementary material

Below is the link to the electronic supplementary material.

Supplementary Material 1

## Data Availability

Enquiries for further data can be directed to the corresponding author.
