# Supplementary material for: Comparisons of treatment performance and therapy sequences in neuroendocrine neoplasms using progression-free survival ratios
Source: Eur J Nucl Med Mol Imaging. 2025 Jun 21;53(1):263–74. doi: 10.1007/s00259-025-07411-y (PMC12660416; doi:10.1007/s00259-025-07411-y)

Supplementary data for:

Comparisons of treatment performance and therapy sequences in neuroendocrine neoplasms using progression-free survival ratios

Table S1. Number of treatments.

**Type**	**NEC**	**NET G1/G2**	**NET G3**	**TC/AC**	**Sum**
**PRRT**	2	86	9	17	114
**SSA**	NA	83	3	12	98
**Other**	18	37	8	3	66
**Everolimus**	4	34	3	18	59
**CAPTEM**	8	8	15	13	44
**Platinum/etoposide**	27	5	6	6	44
**Re-PRRT**	NA	33	3	2	38
**FOLFOX/FOLFIRI**	15	1	3	3	22
**Total**	74	287	50	74	485

The type 'other' comprises the therapies ACO (doxorubicin/cyclophosphamide/vincristine, n=4), carboplatin/docetaxel (n=1), EPICO (epirubicin/cyclophosphamide/vincristine, n=2), FOLFOX + bevacizumab (n=1), interferon + SSA (n=3), irinotecan mono (n=1), lanreotide + temozolomide (n=11), lenvatinib (n=4), nivolumab (n=1), pembrolizumab (n=3), PRRT + capecitabine (n=4), PRRT + CAPTEM (n=2), spartalizumab (n=4), STZ/5-FU (streptozotocin/5-fluorouracil, n=6), sunitinib (n=8), temozolomide mono (n=5), topotecan (n=4), and XELIRI (capecitabine/irinotecan, n=2).

With regards to treatment protocols, 106/110 patients with PRRT had received [^{177}Lu]Lu-DOTA-TATE (96%, excluding 4 NA). Of those, 85 (80%) had 4 cycles (median cumulative activity was 29,7 GBq, the range 13,3–30,6 GBq), eight had 3 cycles (median: 22,2 GBq, range: 18,5–22,9 GBq), seven had 2 cycles (median: 14,9 GBq, range: 11,1–15,1 GBq), four had one cycle (median: 7,5 GBq), and two had 6 cycles (average: 44,8 GBq). Other applied radiopharmaceuticals included [^{90}Y]Y-DOTA-TOC or [^{177}Lu]Lu-DOTA-TOC or protocols were mixed. Overall, 23 patients had Re-PRRT with 1-4 cycles of [^{177}Lu]Lu-DOTA-TATE (except one case with 2x [^{177}Lu]Lu-DOTA-TOC and 2x [^{177}Lu]Lu-DOTA-TATE), six had 2-4 cycles of Re-PRRT twice, and one patient had retreatment with 2-3 cycles thrice. Systemic therapies were applied at standard doses with dose reductions as necessary.

Figure S1. Therapy sequences with the corresponding progression-free survival intervals for NET G1/G2 and TC/AC (A1-A2), NET G3 (B), and NEC patients (C).

Caption: PRRT, peptide receptor radionuclide therapy. SSA, somatostatin analog. EVE, everolimus. CT, capecitabine/temozolomide. PE, platinum/etoposide. RP, Re-PRRT. FF, FOLFOX/FOLFIRI. OTH, other therapies.

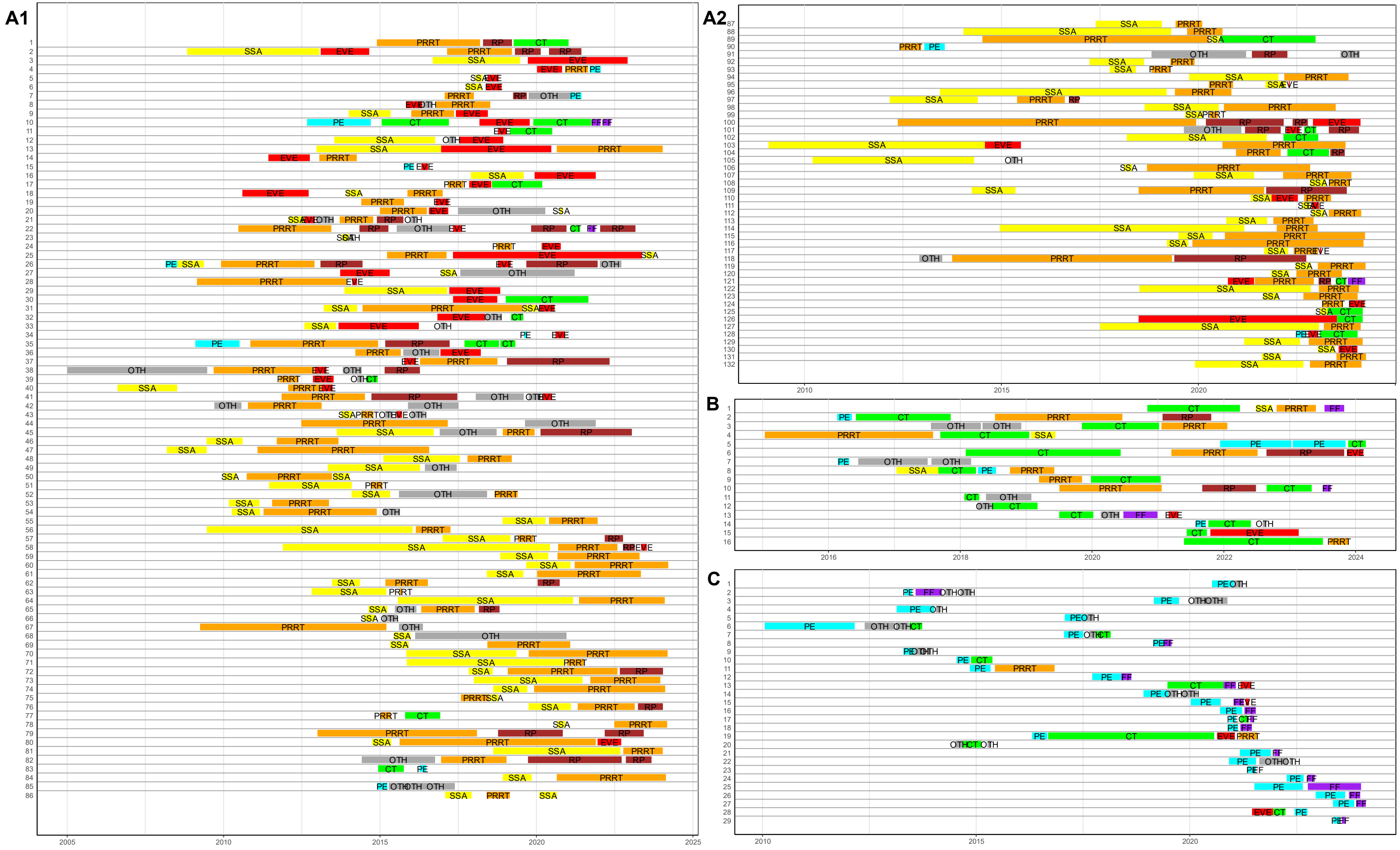

Figure S2. Sankey diagram of treatment sequencing in NET G3 (A) and NEC (B).

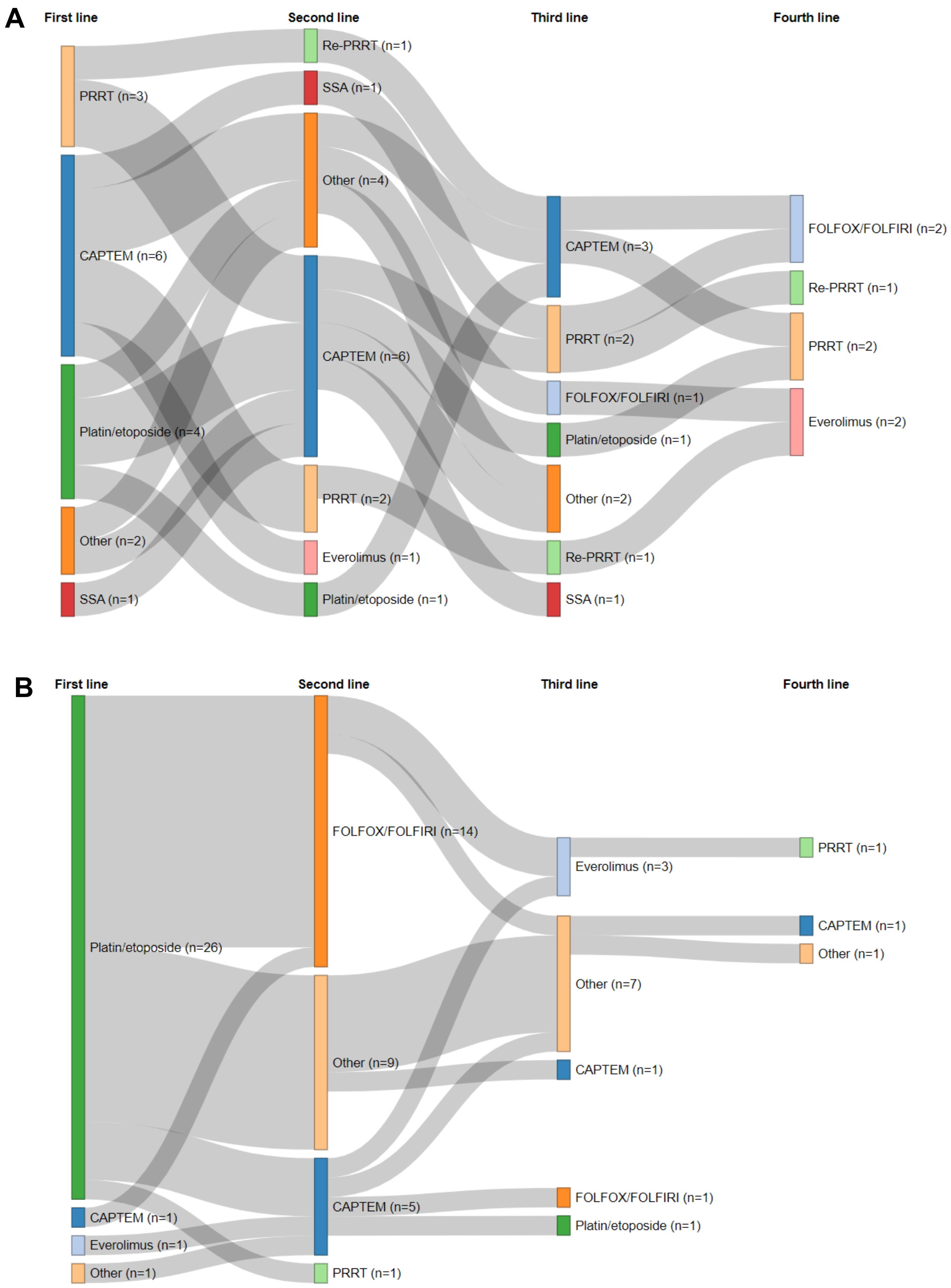

Figure S3. Kaplan-Meier analysis of PFS from first-line palliative treatment initiation in patients with NET G1/G2 or TC/AC.

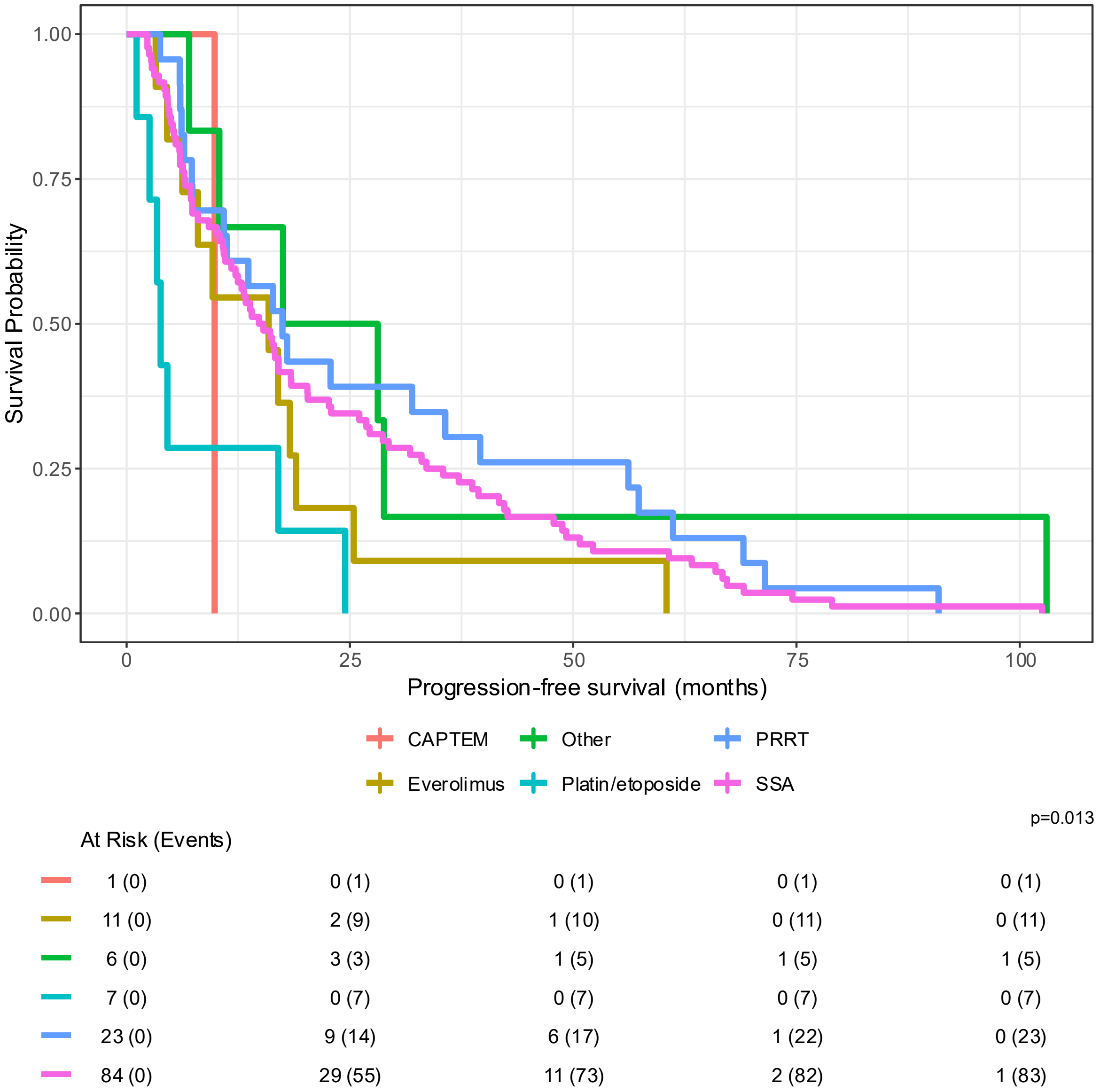

Figure S4. Kaplan-Meier analysis of PFS from second-line palliative treatment initiation in NET G1/G2 and TC/AC.

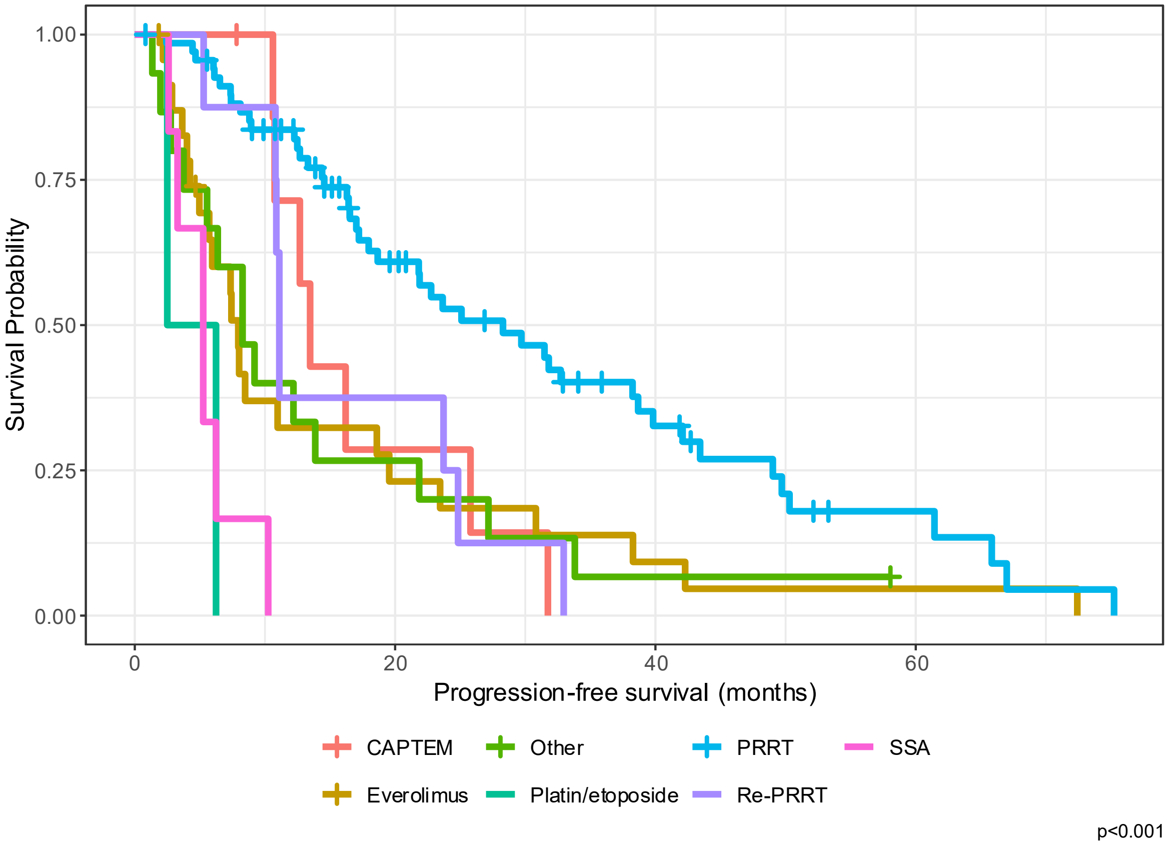

At Risk (Events)

	0	20	40	60
	8 (0)	2 (5)	0 (7)	0 (7)
	24 (0)	5 (17)	2 (20)	1 (21)
	15 (0)	4 (11)	1 (14)	0 (14)
	2 (0)	0 (2)	0 (2)	0 (2)
	69 (0)	32 (24)	13 (37)	4 (42)
	8 (0)	3 (5)	0 (8)	0 (8)
	6 (0)	0 (6)	0 (6)	0 (6)

Figure S5. Spearman correlation between PFS1 and PFS2. Only patients who were progressive in the second line are considered (n=150).

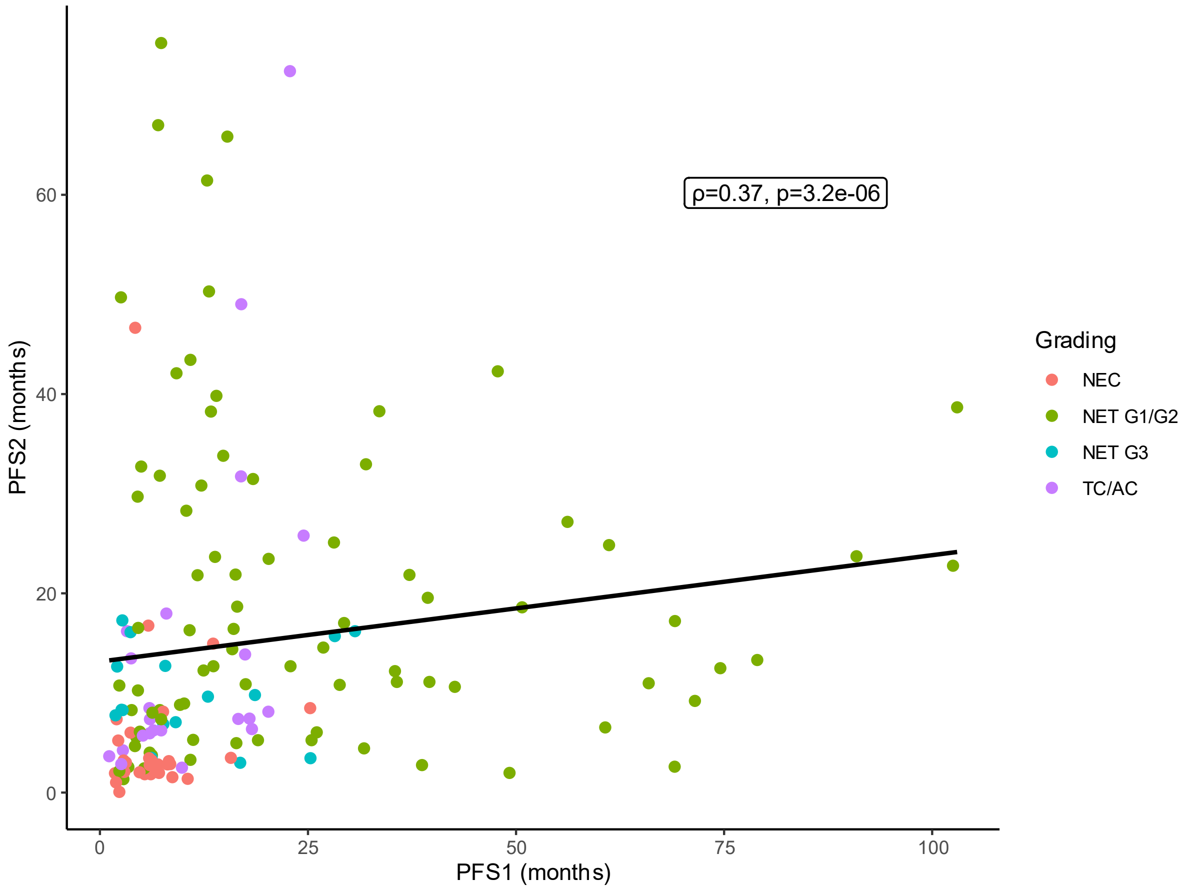

Figure S6. Correlation between PFS1 and PFS2 using partially censored data of all included patients (n=177).

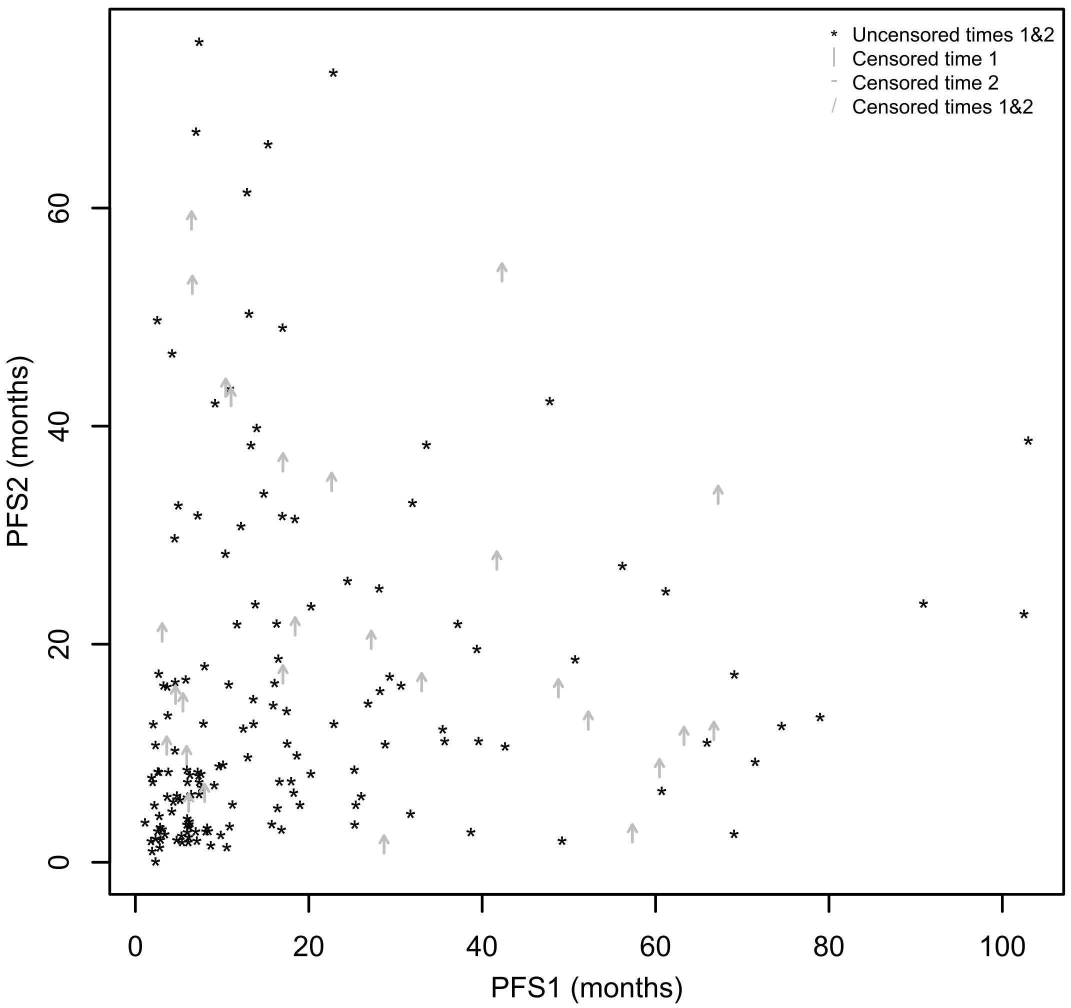

Figure S7. PFS ratios for the sequence SSA to everolimus in individual patients with NET G1/G2 or TC/AC (A), the distribution of these PFS ratios (B), and a Kaplan-Meier (KM) plot showing survival curves for the previous and subsequent treatment (C).

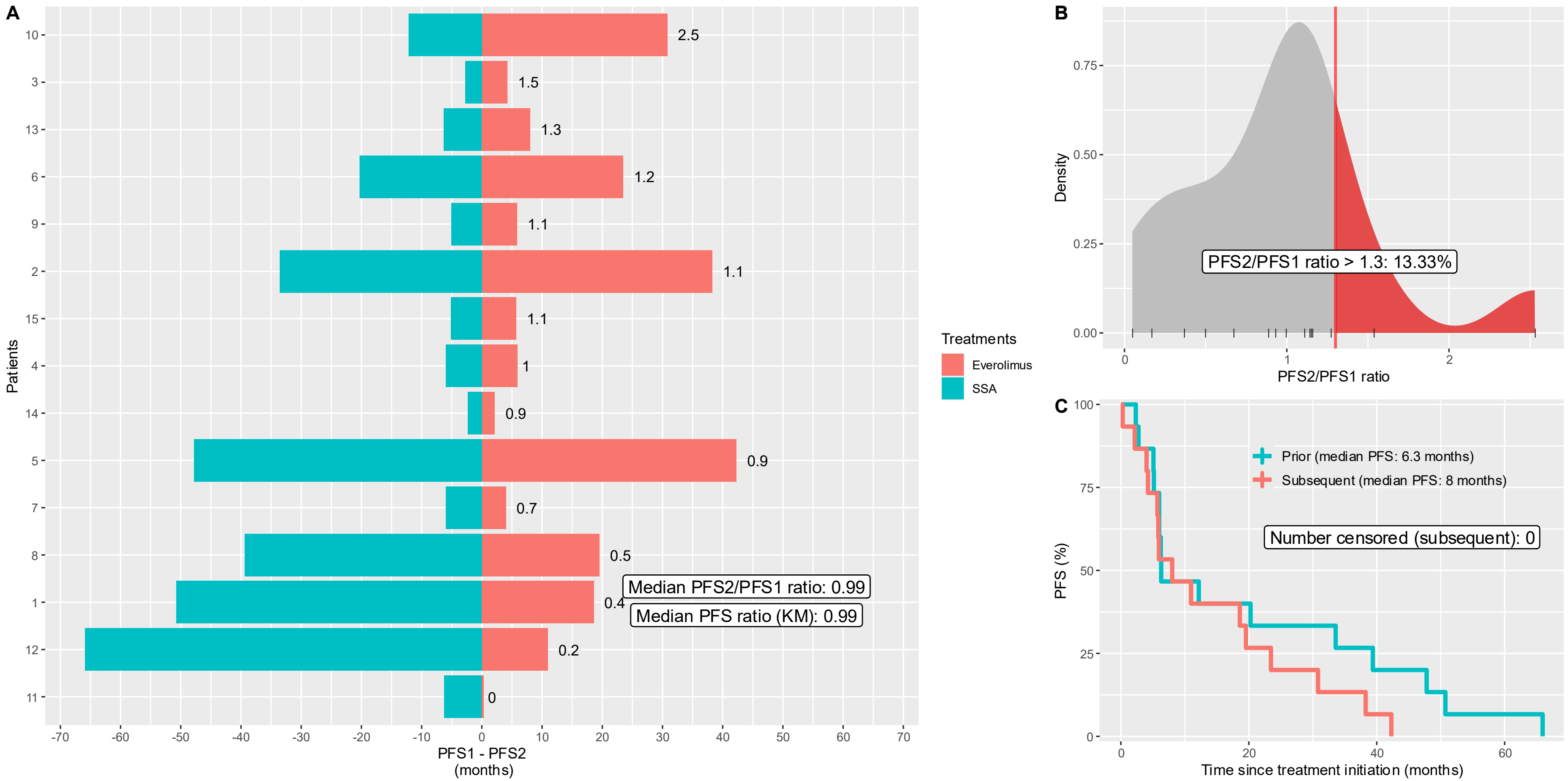

Figure S8. PFS ratios for the sequence PRRT to everolimus in individual patients with NET G1/G2 or TC/AC (A), the distribution of these PFS ratios (B), and a Kaplan-Meier (KM) plot showing survival curves for the previous and subsequent treatment (C).

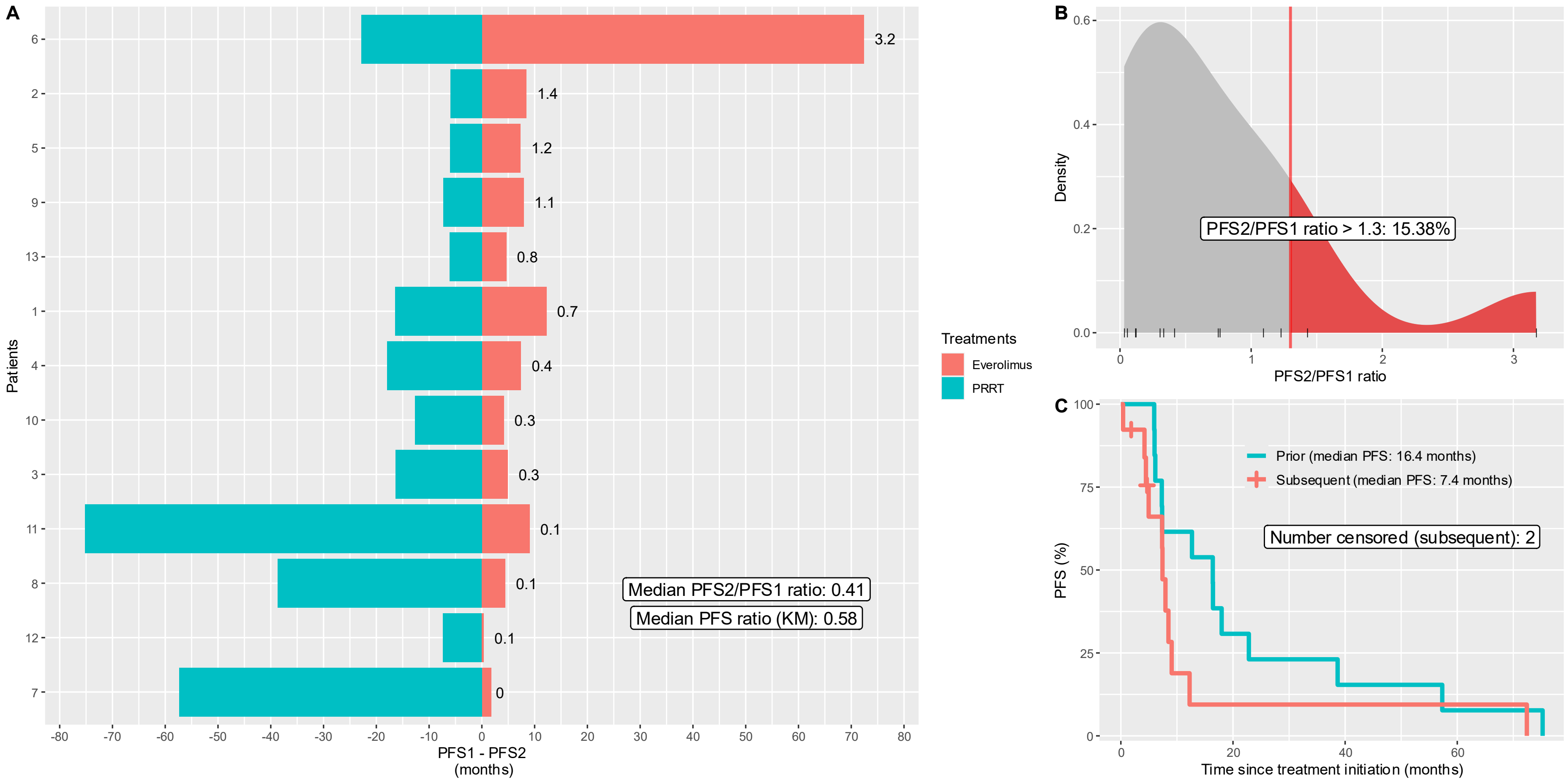

Figure S9. PFS ratios for the sequence platinum/etoposide to FOLFOX/FOLOFIRI in individual NEC patients (A), the distribution of these PFS ratios (B), and a Kaplan-Meier (KM) plot showing survival curves for the previous and subsequent treatment (C).

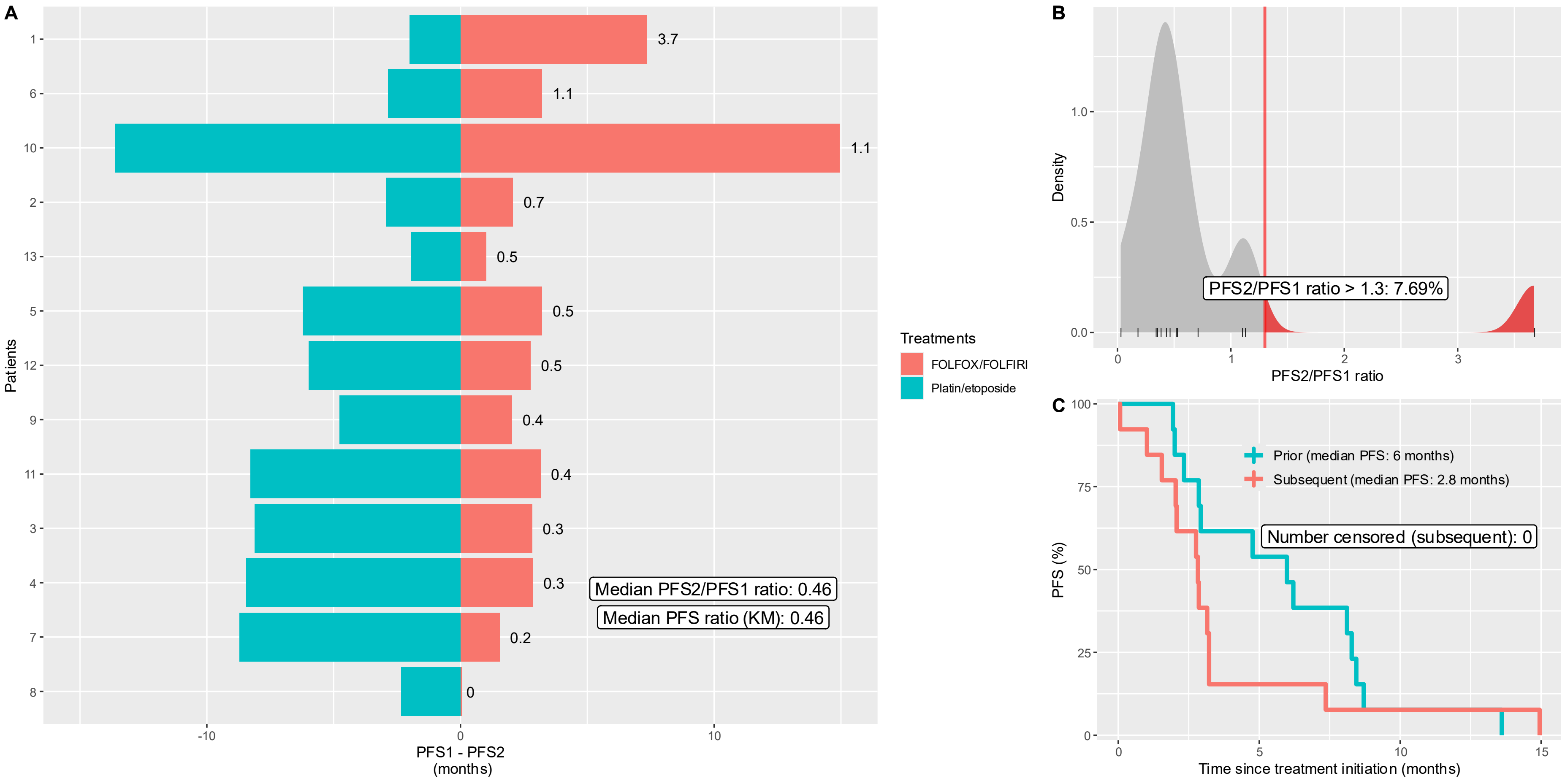

Figure S10. PFS ratios for the sequence platinum/etoposide to CAPTEM in individual patients with NET G3 (A) or NEC (B).

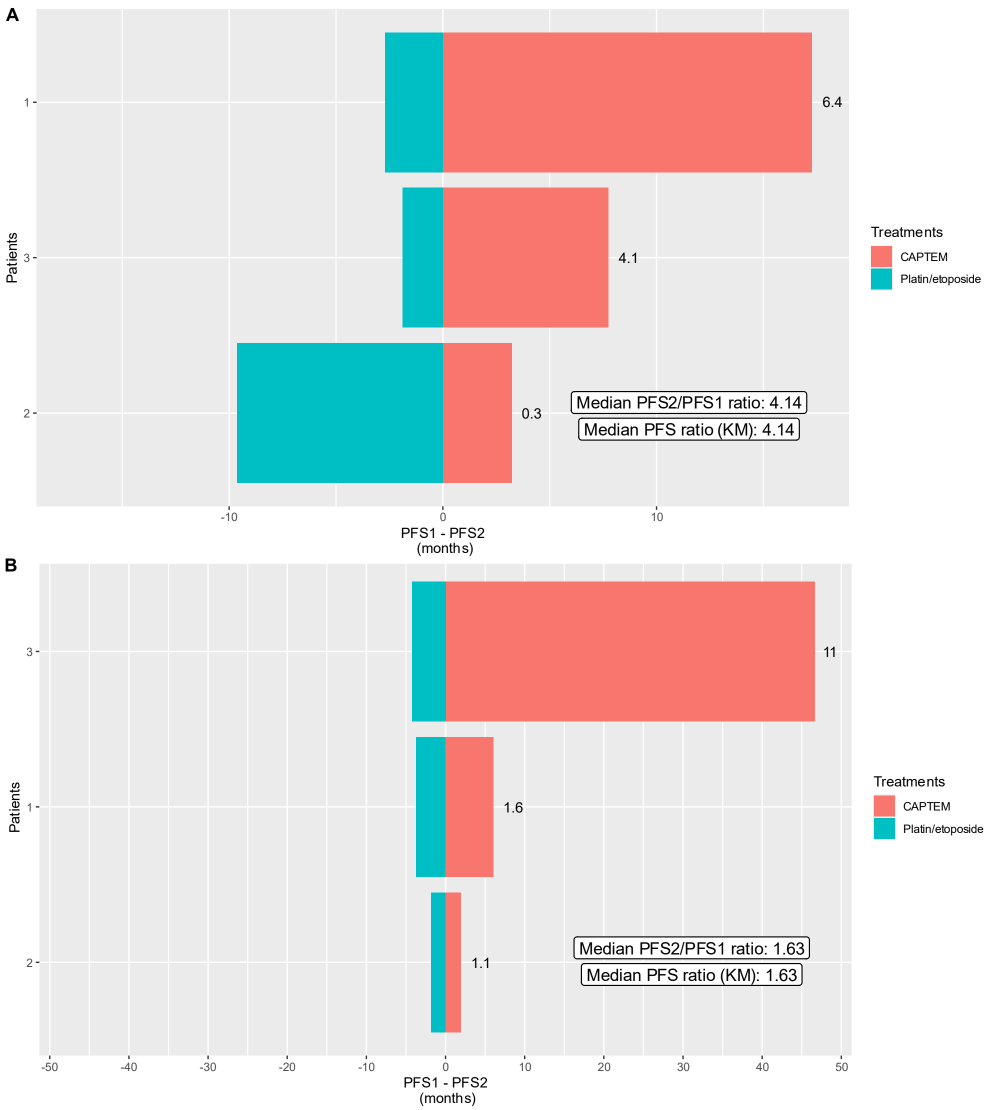

Figure S11. Kaplan-Meier analysis of overall survival (OS) following first-line treatment start according to grading.

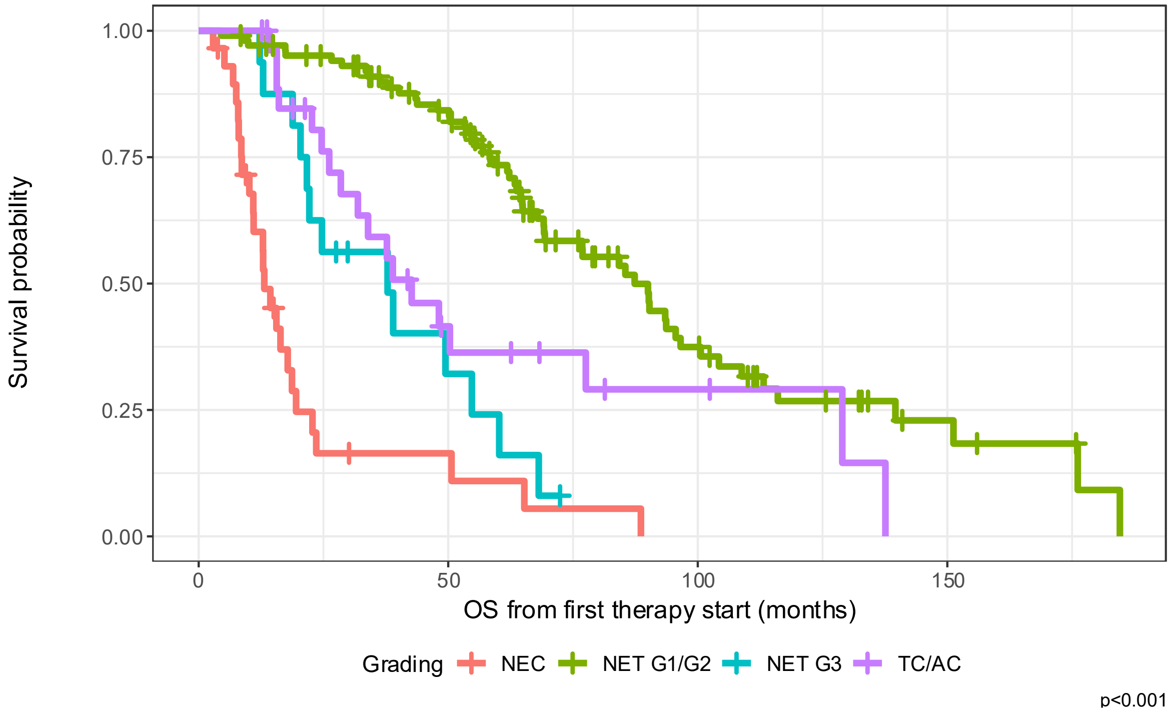

At Risk (Events)

NEC	29 (0)	3 (22)	0 (25)	0 (25)
NET G1/G2	104 (0)	74 (15)	21 (47)	5 (53)
NET G3	16 (0)	4 (10)	0 (13)	0 (13)
TC/AC	28 (0)	8 (14)	3 (16)	0 (18)

Figure S12. Kaplan-Meier analysis of overall survival (OS) after initiation of different second therapies in patients with NET G1/G2 or TC/AC.

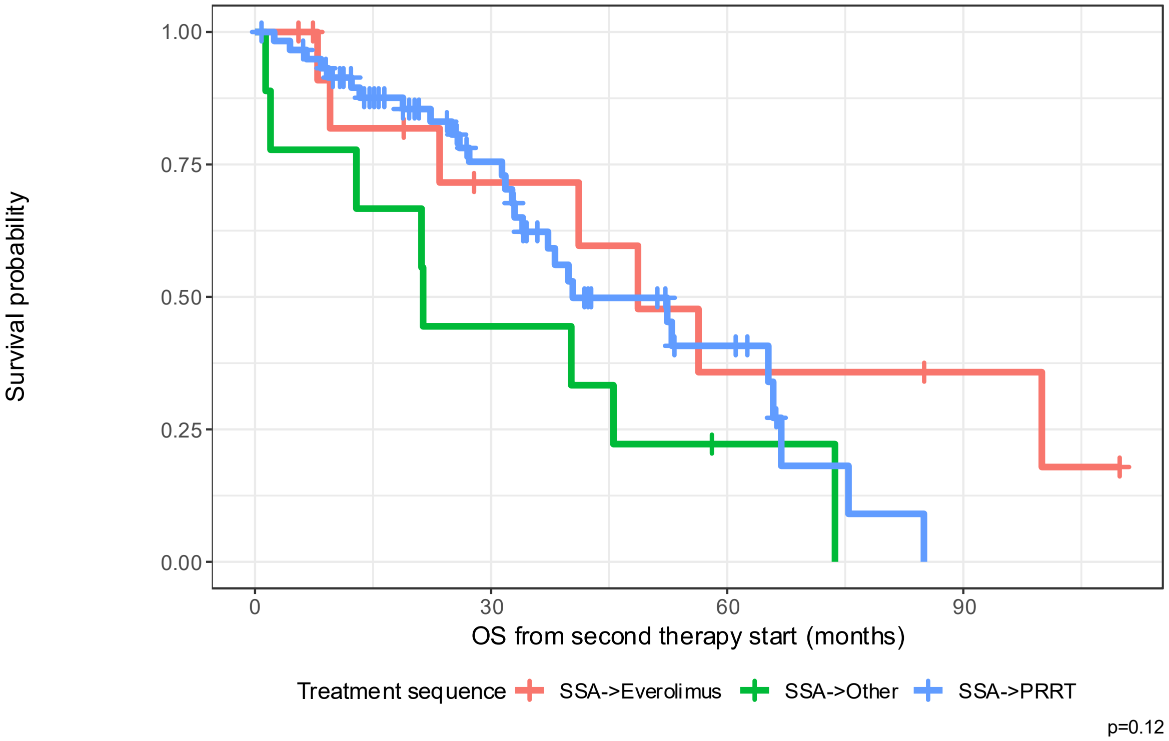

At Risk (Events)

SSA->Everolimus	13 (0)	6 (3)	3 (6)	2 (6)
SSA->Other	9 (0)	4 (5)	1 (7)	0 (8)
SSA->PRRT	60 (0)	29 (12)	8 (23)	0 (28)

Figure S13. Cox regression analysis of overall survival (OS) following second-line treatment start in patients with NET G1/G2 or TC/AC.

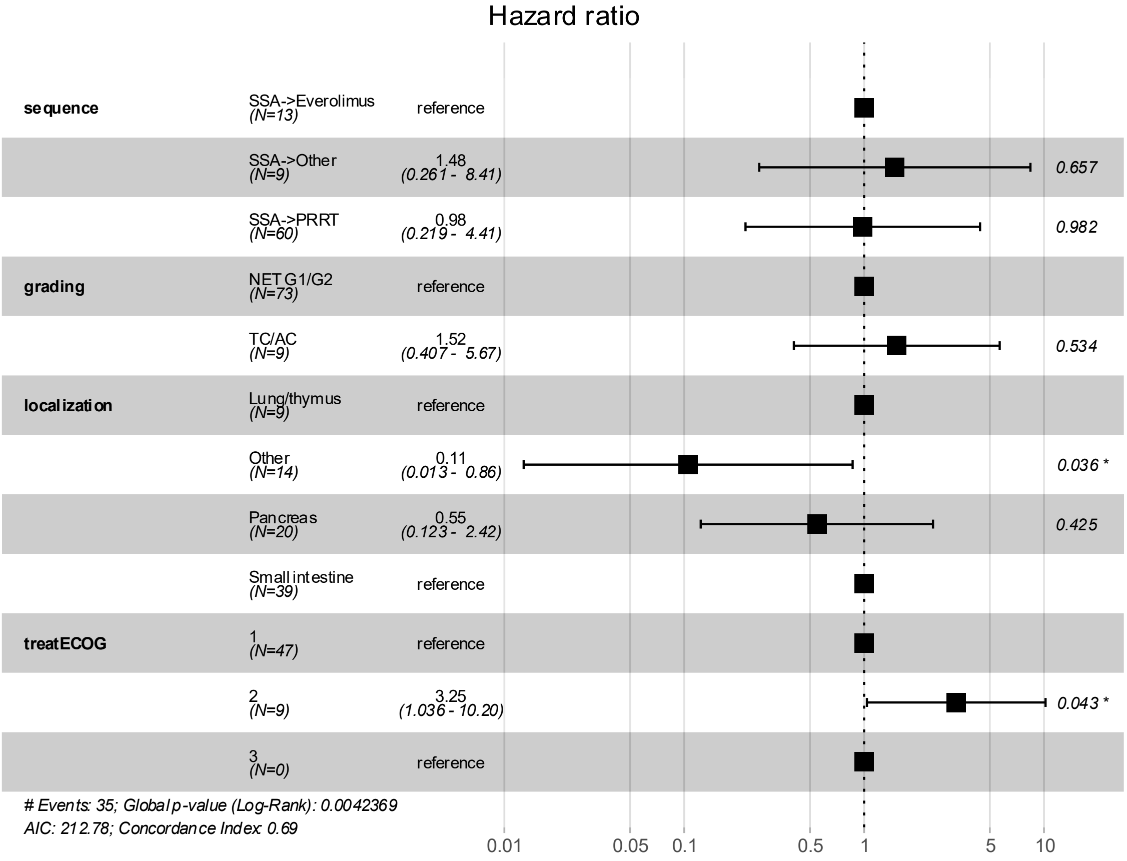

Supplement: Supplementary file 1 — Supplementary Material 1 [file 259_2025_7411_MOESM1_ESM.pdf]
